# Editorial: Management of Bone Disorders in Children

**DOI:** 10.3389/fendo.2021.725655

**Published:** 2021-07-05

**Authors:** Janet L. Crane, Madhusmita Misra

**Affiliations:** ^1^ Division of Pediatric Endocrinology, Johns Hopkins University School of Medicine, Baltimore, MD, United States; ^2^ Division of Pediatric Endocrinology, Massachusetts General Hospital and Harvard Medical School, Boston, MA, United States

**Keywords:** bone disorders, osteoporosis management, X-linked hypophosphatemia, oral contraception, giant cell tumor, vitamin d deficient rickets, denosumab, burosumab

Poor bone health in children increases the risk for fracture. Further, conditions that impact the structural framework of bone and its mineralization may lead to deformed bones. The childhood and adolescent years are a critical period for bone accrual with 50% of peak bone mass being achieved during the adolescent years, and more than 80% by 18 years (1). Because peak bone mass is a key determinant of future bone health, impaired bone accrual during childhood and adolescence may increase the long-term risk of fracture. Non-modifiable determinants of bone accrual include genetic factors and diseases, whereas modifiable determinants include nutritional status, mechanical loading, hormones, chronic systemic diseases and chronic use of certain medications. In order to optimize bone health in a pediatric population and reduce both immediate and future fracture risk as well as the risk for longstanding bone deformity, it is essential to manage these disorders in a timely and effective manner.

Such management can be complex and challenging. When the cause is modifiable, the first step may involve nutritional recommendations, attention to mechanical loading of bones, optimizing pubertal status, and addressing underlying chronic disease/s. In recent times, data have emerged regarding pharmacological interventions for certain conditions, particularly in children with genetic diseases causing impaired bone mineral metabolism, those receiving chronic glucocorticoids, and children with malignancies. This Research Topic covers the management of children suffering from some of these conditions, covered by experts in the field.

Use of hormonal contraception is now widespread, and while its benefits are well known, there is concern regarding its effects on bone, particularly during peak bone accrual. Bachrach reports deleterious effects of combined oral contraceptives (COCs) and intramuscular depot medroxyprogesterone acetate (DMPA) on bone accrual in adolescents; COCs by reducing insulin like growth factor-1 (IGF-1) levels, and DMPA by reducing estrogen levels. In contrast, the few studies that have examined effects of progestin implants and progestin releasing IUDs (long acting reversible contraception) on bone do not demonstrate deleterious effects. Some studies, but not all, suggest a higher risk of fracture among COC users during adolescence, and a higher risk of fracture is reported in those using DMPA. Bachrach concludes that further studies are necessary to clarify the impact of dose, duration of use, and route of administration of hormonal contraception on bone in adolescents.

The paper by Allaway et al. expands on this theme and examines whether effects of oral and vaginal contraceptives on bone formation in young women are mediated *via* the growth hormone (GH)-IGF-1 axis. The authors use the IGF-1 generation test to test their hypotheses and report suppression of the peak IGF-1 response following recombinant GH (rGH) administration in the COC group, but not the contraceptive vaginal ring (CVR) or control groups, suggesting route dependency of this effect. They also report suppression of the bone formation marker, N-terminal propeptide of Type 1 procollagen, in both contraceptive groups, but not in controls, suggesting a route independent effect. The authors conclude that larger studies are necessary at all ages to expand on these results. Further, studies comparing the COC and CVR to the contraceptive patch are necessary.

The reviews by Schindeler et al. and Boyce et al. focus on disorders of phosphate metabolism. While our understanding of these disorders has increased markedly over the past two decades, much work is necessary to optimize management. Schindeler et al. review data regarding treatment with burosumab (a fibroblast growth factor 23 (FGF23) neutralizing antibody) for X-linked hypophosphatemia (XLH) and conditions such as tumor induced osteomalacia (TIO), epidermal nevus syndromes (ENS), and cutaneous skeletal hypophosphatemia syndrome. Short-term studies suggest that burosumab is both efficacious and safe in adults and children with XLH, TIO and ENS. However, studies are necessary to ensure that there is not an increased longer-term risk for nephrocalcinosis and cardiac calcifications with burosumab. The review by Boyce et al. focuses on the pathogenesis, presentation and management challenges of hyperphosphatemic familial tumoral calcinosis (HFTC), a rare disorder caused by either a deficiency or resistance to FGF23. The review describes the genetic basis for HFTC and also describes an acquired autoimmune form of hyperphosphatemic tumoral calcinosis. The paper discusses current approaches to therapy including a low phosphate diet, phosphate lowering, anti-inflammatory and anti-mineralization strategies, surgical resection, and physical and occupational therapy. Overall, no definitive therapies have been identified for this condition thus far.

While vitamin D deficient rickets (VDDR) has been largely prevented/cured by supplementation, Levine discusses treatment of rare genetic defects in the vitamin D pathway and difficulties in management. VDDR can be classified as: 1) the inability to either complete 25-hydroxylation or 1-hydroxylation of calciferols; 2) resistance to 1,25 (OH)_2_D from mutations in the vitamin D receptor (VDR) gene or the presence of a ribonucleoprotein that interferes with VDR-DNA interaction; or 3) excessive inactivation of vitamin D metabolites. The goal of treatment for these various forms is similar, and includes normalization of bone metabolism biochemistries (particularly calcium and PTH) while monitoring for rickets and nephrocalcinosis, by replacing the deficient vitamin D metabolite and aggressive vitamin D and calcium supplementation.

Glucocorticoid-induced osteoporosis in the pediatric population is unique with early vertebral compression fractures that have the ability to reshape depending on growth potential. Ward outlines a blueprint for monitoring osteoporosis in children treated with glucocorticoids and provides guidance as to whether osteoporosis therapy may be indicated, discussing potential use of bisphosphonates as the current standard of care versus alternative potential agents that remain unstudied, including denosumab, teriparatide, and romosozumab.

Ferriero et al. report on the safety and efficacy of denosumab in the treatment of four children with multiple giant cell lesions of the jaw with Noonan syndrome. All children had improvement in clinical and radiographic symptoms. Adverse effects were as expected and included hypocalcemia during treatment and rebound hypercalcemia following cessation of denosumab. One child also experienced significant arthralgia on treatment independent of calcium dysregulation.

Overall, the Research Topic covers a wide array of pediatric bone health conditions and treatment approaches that will become an important resource for professionals managing children and adolescents.

## Author Contributions

All authors listed have made a substantial, direct, and intellectual contribution to the work and approved it for publication.

## Funding

JLC received funding support from the US National Institutes of Health (R03AR073939, R01AR078793). MM received funding support from the US National Institutes of Health (R0IHD060827, K24HD071843).

## Conflict of Interest

MM has served on the scientific advisory board of Abbvie and Ipsen, and as a consultant for Abbvie and Sanofi.

The remaining author declares that the research was conducted in the absence of any commercial or financial relationships that could be construed as a potential conflict of interest.
